# Metal‐Free Photocatalysts for Conversion of H_2_O into Hydrogen Peroxide

**DOI:** 10.1002/cssc.202201514

**Published:** 2022-11-04

**Authors:** Qiao Wang, Xin Ying Kong, Yongye Wang, Li Wang, Yingping Huang, Hui Li, Tianyi Ma, Liqun Ye

**Affiliations:** ^1^ College of Materials and Chemical Engineering Key Laboratory of Inorganic Nonmetallic Crystalline and Energy Conversion Materials China Three Gorges University Yichang 443002 P. R. China; ^2^ Division of Chemistry and Biological Chemistry School of Chemistry, Chemical Engineering and Biotechnology Nanyang Technological University 21 Nanyang Link 637371 Singapore; ^3^ Engineering Research Center of Eco-environment in Three Gorges Reservoir Region Ministry of Education China Three Gorges University Yichang 443002 P. R. China; ^4^ School of Science RMIT University Melbourne VIC 3000 Australia

**Keywords:** artificial photosynthesis, H_2_O_2_, organic semiconductors, photoabsorption, photocatalysis

## Abstract

Hydrogen peroxide (H_2_O_2_) is an important green oxidizing agent for environmental protection and chemical production. In comparison to the traditional anthraquinone method, photosynthesis is a green and energy‐saving process for H_2_O_2_ production. To improve the stability and practical application value of the H_2_O_2_ synthesized by photocatalysis, the H_2_O_2_ photosynthesis should be conducted in pure water without involving any sacrificial reagents. In this regard, organic semiconducting catalysts pose as a suitable candidate for photocatalytic H_2_O_2_ synthesis owing to their metal‐free nature to prevent H_2_O_2_ decomposition by the metal ions. In this Perspective, the H_2_O_2_ photosynthesis history is firstly introduced, followed by a review of the organic semiconductor photocatalysts reported to date. Finally, the main problems to thwart the advances of current pure H_2_O‐to‐H_2_O_2_ photosynthesis are discussed, followed by proposed solutions to address these issues in order to pave new ways for the development of highly efficient metal‐free organic photocatalysts for sustainable pure H_2_O‐to‐H_2_O_2_ conversion.

## Graphical abstract

**Photocatalysis for H_2_O_2_**: Metal‐free organic semiconductors are next‐generation photocatalysts for the synthesis of H_2_O_2_ from water and molecular oxygen. Photoabsorption ability, separation efficiency of photogenerated charge carriers, reaction active sites, and crystallinity of the catalysts are the major factors to affect the yields of H_2_O_2_ photosynthesis. This work provides a useful guidance to the researchers in this field to synthesize highly efficiently organic photocatalysts.

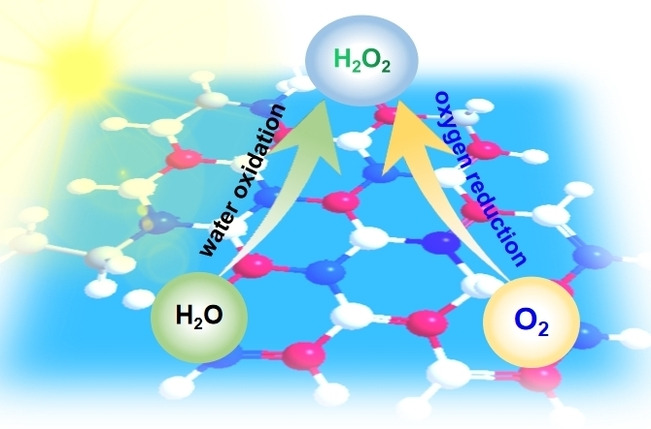

## Introduction

Hydrogen peroxide (H_2_O_2_) is a strong oxidizing agent which possesses antibacterial, antiviral, and disinfectant properties.^[1]^ In addition, it is widely used in various industries including chemical synthesis, textiles and pulp bleaching, wastewater treatments, and electronic applications.^[2]^ Since the decomposition of H_2_O_2_ will only produce water (H_2_O) without other hazardous products, H_2_O_2_ is regarded as an environmentally friendly chemical.^[3]^ Although anthraquinone oxidation is the most commonly employed method for the production of H_2_O_2_,^[4]^ there are still some unsettling problems in this method that should not be neglected. For instance, the anthraquinone oxidation involves a sequential process including hydrogenation, oxidation, extraction, and purification, which require large inputs of energy, and it is potentially dangerous.^[5]^ Moreover, the solvents used in the production process may lead to severe environmental pollution, which conflicts with the original idea of green chemistry. In this regard, photocatalysis, a process capable of converting free, inexhaustible solar energy into chemically valuable substances with the aid of a suitable photocatalyst, is a remarkable approach to replace the anthraquinone process.^[6]^ Through photocatalytic route, H_2_O and O_2_ are the only raw materials needed with the presence of sunlight to provide energy for the formation of H_2_O_2_.^[7]^ For example, it has been shown that H_2_O_2_ can be photosynthesized from seawater directly in air by the hydrolysis of lignin.^[8]^ This is a simple process that can take place at mild conditions (room temperature and atmospheric pressure), and more importantly, it does not produce harmful substances to contaminate the environment.^[9]^ Therefore, the photocatalytic process is in good agreement with the concept of green chemistry.

**Scheme 1:**
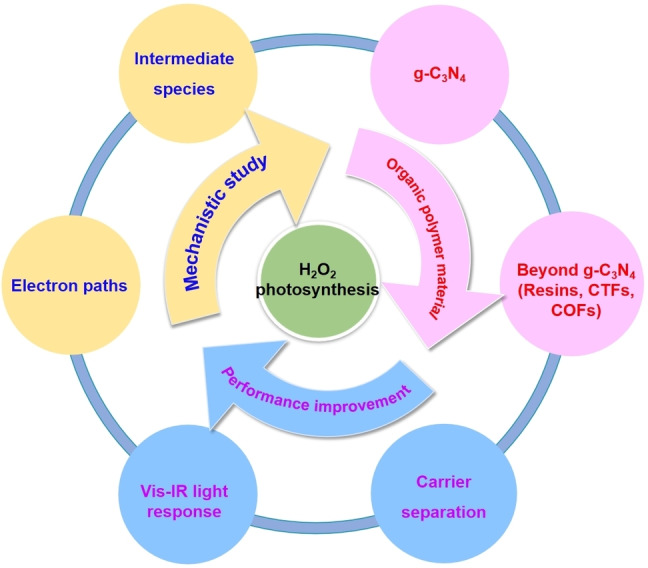
Organic semiconductor photocatalyst for H_2_O_2_ photosynthesis.

In order to prevent H_2_O_2_ decomposition by metal and ultraviolet (UV) light,^[10]^ visible‐light‐responsive metal‐free organic semiconductors are the current main focus regarding photocatalysts for achieving H_2_O_2_ photosynthesis in high concentration. However, organic semiconductors often exhibit poor separation of photogenerated charge carriers, and hence, sacrificial reagents such as ethanol and methanol are usually added to consume the photoinduced holes to thwart the electron–hole pairs recombination for enhanced photocatalytic efficiency. However, the residual sacrificial reagents will severely affect the stability and purity of the H_2_O_2_ produced, thereby affecting the practical application value of the H_2_O_2._ Therefore, many organic semiconducting polymers,^[11]^ such as graphitic carbon nitride (g‐C_3_N_4_), resins, covalent triazine frameworks (CTFs), and covalent organic framework (COFs), were modified by constructing opportune active sites at the molecular level to photosynthesize high concentration of H_2_O_2_ from pure water without any sacrificial reagents. In this Perspective, the state‐of‐the‐art organic semiconductor photocatalysts with various modification approaches are reviewed (Scheme 1). We also raise the main issues that currently hinder the practical applications of H_2_O_2_ photosynthesis, and lastly, we propose some potential solutions to solve these issues in order to bring the research community a step closer towards the commercialization of photocatalytic H_2_O_2_ production from pure water.

## A Brief History of H_2_O_2_ Photosynthesis

Solar light‐driven production of H_2_O_2_ from water was first proposed by Baur and Neuweiler in 1921 by employing ZnO as a catalyst to produce H_2_O_2_ in the presence of glycerol and glucose.^[12]^ Since then, various inorganic materials such as TiO_2_, WO_3_, CdS, and so on have been studied for photocatalytic production of H_2_O_2_.^[13]^ However, it was found that the metal element composition often decomposes the generated H_2_O_2_, leading to a low solar‐to‐chemical conversion (SCC) efficiency. In 2014, Shiraishi et al. firstly reported metal‐free g‐C_3_N_4_ photocatalysts for H_2_O_2_ photosynthesis,^[14]^ Following this, the scope of application of organic polymer semiconductors was extended quickly. After years of study, researchers found that g‐C_3_N_4_ suffers from intrinsic low photogenerated charge carrier separation efficiency, narrow light absorption range, low crystallinity, and exhibits single active sites, which greatly limits the photocatalytic performance of H_2_O_2_ synthesis. Therefore, finding new metal‐free photocatalysts and the relevant modification methods to further improve the photocatalytic performance is the topic of current study.

## Organic Semiconductors for H_2_O_2_ Photosynthesis

### g‐C_3_N_4_

**Figure 1:**
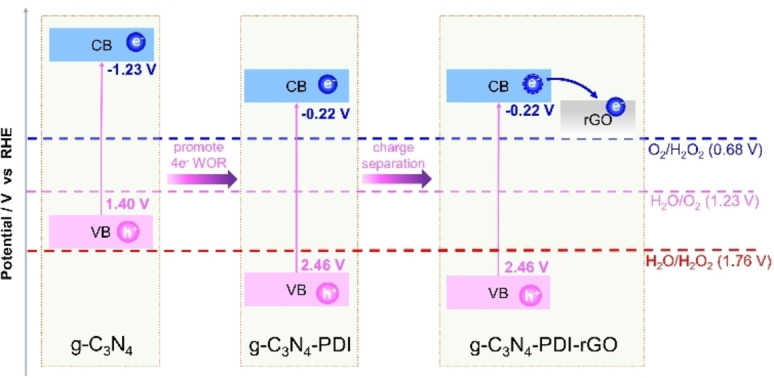
Improving the photocatalytic activity of H_2_O_2_ synthesis over g‐C_3_N_4_ through strategic coupling.

**Figure 2:**
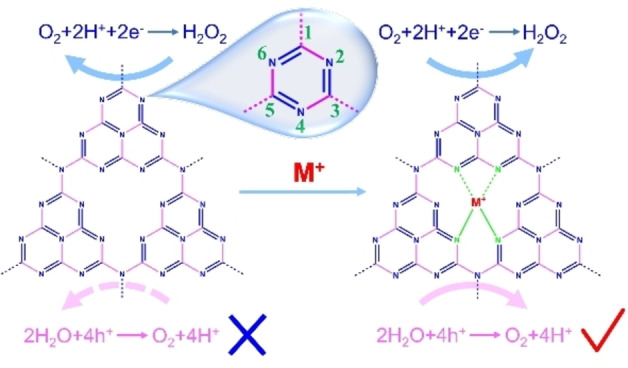
Single atom engineering to improve the water oxidation capability of g‐C_3_N_4._

g‐C_3_N_4_ is an organic layered polymer composed of C and N elements with high thermal and chemical stability.^[15]^ Owing to its excellent chemical stability and unique electronic band structure, it is widely studied in photocatalytic degradation of pollutants, hydrogen and oxygen production from water splitting, and organic synthesis.[^16^, ^17^] In 2014, g‐C_3_N_4_ was first studied for H_2_O_2_ photosynthesis in ethanol solution.^[14]^ During the photocatalytic reaction over g‐C_3_N_4_, the formed internal peroxides prevented one‐electron reduction of O_2_. However, the H_2_O_2_ yield over g‐C_3_N_4_ was still very low, only 30 μmol after 12 h of light illumination. The low yield could be attributed to several reasons: (1) the intrinsic weak absorption of visible light; (2) low separation efficiency of the photogenerated charge carriers; (3) low crystallinity of g‐C_3_N_4_ inhibiting charge transfer. Therefore, incessant research efforts were devoted to improving the efficiency of g‐C_3_N_4_ for H_2_O_2_ photosynthesis. In order to enhance the water oxidation activity for H_2_O_2_ photosynthesis in pure water, g‐C_3_N_4_ was compounded with homophthalic diimide (PDI).^[18]^ The valence band position of the compound was positively shifted to promote the oxidation of water for the generation of H_2_O_2_, while ensuring that the conduction band position can still effectively reduce oxygen, thereby forming a dual pathway to generate H_2_O_2_ from pure water (Figure 1). Theoretical study revealed that the electrons and holes are respectively generated at C1, N4 and N2, N6 positions under solar excitation (the specific structure is shown in Figure 2). Particularly, the holes oxidize H_2_O to generate O_2_, whereas two electrons reduce O_2_ to generate internal peroxide, which then convert into H_2_O_2_ by protonation. The whole process effectively suppresses the single‐electron and four‐electron reduction of O_2_. Subsequently, reduced graphene oxide (rGO) was further hybridized with C_3_N_4_/PDI,^[19]^ and the incorporation of rGO greatly improved the separation of photogenerated charge carriers (Figure 1). By comparing the photocatalytic performance, the apparent quantum efficiency (AQE) at 420 nm was determined to be 6.1 %, and the average SCC efficiency was enhanced to 0.20 %. By further adding boron nitride (BN) to the g‐C_3_N_4_/PDI/rGO system, the g‐C_3_N_4_/PDI/rGO/BN composite exhibited an average SCC efficiency of 0.27 %.^[20]^ Interestingly, the researchers found that the electron–hole recombination efficiency of the material was greatly inhibited by the introduction of aryl amino groups to g‐C_3_N_4_, while promoting the oxygen reduction reaction (ORR), which could produce 2.0 mm h^−1^ H_2_O_2_ in the presence of household LED.^[21]^

Besides, single atoms, which serve as the catalytic reactive sites, are often used in g‐C_3_N_4_ for H_2_O_2_ photosynthesis from pure water (Figure 2). It was reported that Co single atom and anthraquinone (AQ) can be used as the oxidation and reduction co‐catalysts of g‐C_3_N_4_, respectively.^[22]^ The spatial separation of the redox centers can effectively inhibit the complexation of electron–hole pairs while increasing the reaction active sites, rendering a H_2_O_2_ yield of 60 μm h^−1^ over Co_1_/AQ/C_3_N_4_. In addition, when Sb single atom is loaded on g‐C_3_N_4_,^[23]^ the conduction band electrons accumulate on the Sb sites whereas the holes focus on the N atoms near the single Sb atom, thereby suppressing the recombination of electron–hole pairs. With this elegant design, even in the absence of electron donor, there are still Sb with terminal adsorption conformation ⋅OOH formation, which significantly suppressed the occurrence of undesirable 4e^−^ ORR side reaction, thereby allowing the highly selective occurrence of the 2e^−^ ORR reaction with an average SCC efficiency of 0.61 % for the optimal Sb‐SAPC15. It is challenging to find a suitable modification method for g‐C_3_N_4_ to efficiently improve the photocatalytic efficiency. Therefore, a continuous search for more suitable organic semiconductor materials for H_2_O_2_ photosynthesis is necessary.

### Resins

**Figure 3:**
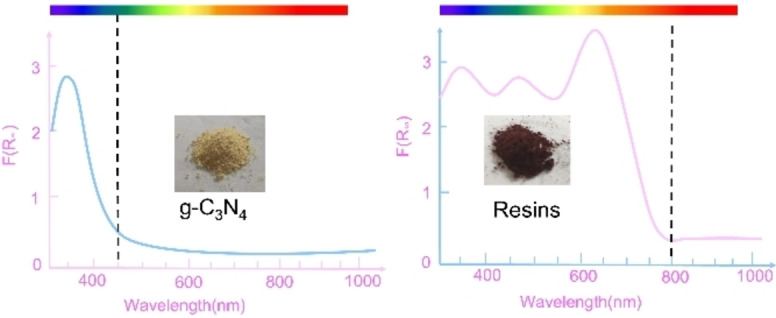
Enhanced visible light to near‐infrared response of resins for enhanced pure H_2_O‐to‐H_2_O_2_ photosynthesis.

Compared to g‐C_3_N_4_, resins usually possess a narrower bandgap that can absorb more visible light to facilitate oxygen reduction and water oxidation (Figure 3). In 2019, resorcinol‐formaldehyde (RF) resin with unique π‐conjugated and π‐stacked benzene–quinoline donor–acceptor (D–A) structure was reported for the first time for H_2_O_2_ photosynthesis in pure water.^[24]^ The narrow bandgap of RF resin greatly promotes oxygen reduction and water oxidation, in which H_2_O_2_ was stably generated with an average SCC yield of 0.5 % without involving any sacrificial reagents. However, a defective valence band structure is present in the RF resin, which limits the transportation of electrons, thus leading to a low photocatalytic efficiency. To overcome the defective valence band structure, polythiophene (P3HT) was doped in the RF resin.^[25]^ It was found that P3HT with suitable highest occupied molecular orbital (HOMO) can be used as a charge transfer polymer for efficient electron migration from the quinone‐type acceptor site, thereby enhancing the water oxidation reaction (WOR) and ORR reactions for generating high concentration of H_2_O_2_ solution with an SCC efficiency of 1.0 %. In addition, monodisperse RF resin microspheres (MRFs) was also studied, in which the mesoporous microspheres can effectively modulate the charge distribution in the nano space to result in an SCC efficiency of 1.1 %.^[26]^ This indicates that the changes in the morphology will lead to the differences in photocatalytic performance. Although resin addresses the issue of narrow forbidden bandwidth of g‐C_3_N_4_, H_2_O_2_ can only be directly generated from the O_2_ reduction through this pathway, whereas no H_2_O oxidation could take place as there is no suitable active site to oxidize H_2_O directly into H_2_O_2_.

### CTF

**Figure 4:**
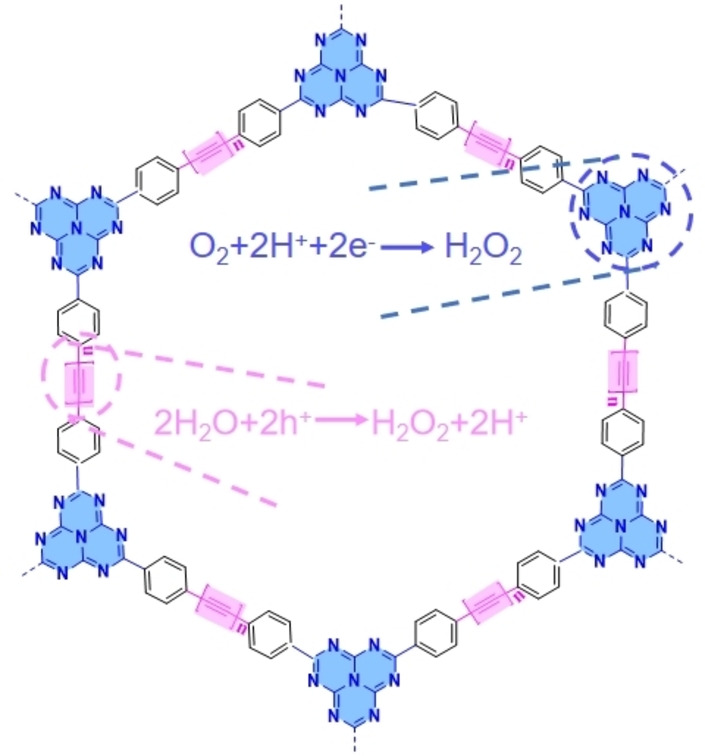
CTFs for H_2_O_2_ photosynthesis via 2e^−^ dual channels process.

CTFs own a large number of oxygen‐reducing active sites (C=N), which is similar to g‐C_3_N_4_. Meanwhile, organic water oxidation sites can also be artificially introduced into CTF through structural modification. Hence, CTF may be an ideal material for H_2_O_2_ photosynthesis via 2e^−^ dual channels. It was reported that the introduction of acetylene and diacetylene can serve as the active sites for WOR (Figure 4). The comparative experiments and theoretical calculations revealed that the conjugated structures of CTF‐EDDBN (4,4′‐(ethyne‐1,2‐diyl)dibenzonitrile) and CTF‐BDDBN (4,4′‐(buta‐1,3‐diyne‐1,4‐diyl)dibenzonitrile) can promote charge separation for the formation of suitable intermediates and create a new pathway for the generation of H_2_O_2_ by 2e^−^ WOR.^[27]^ Although the ORR and WOR pathways can be realized simultaneously for H_2_O_2_ generation, the photogenerated charge carriers tend to recombine at the redox‐active site, which limits the photocatalytic efficiency. Recently, researchers have rationalized the design of novel covalent heptazine frameworks (CHF) with spatially separated redox centers by rationalizing the structure at the molecular level.^[28]^ For instance, s‐heptazine ring, which is connected by three s‐triazine rings, is rich in single atoms and thus is a strong electron‐deficient group that can be used as an active site for O_2_ reduction. From photocatalytic experiments, it exhibited excellent photocatalytic efficiency with an average SCC efficiency of 0.76 %, and the H_2_O_2_ yield over CHF‐DPDA (diphenyldiacetylene) was measured to be 69 μmol h^−1^, which was much higher than that of C_3_N_4_ and CTF‐BDDBN (g‐C_3_N_4_: 2.5 μmol h^−1^; CTF‐BDDBN: 2.9 μmol h^−1^). However, most of the triazine organic semiconductors are in amorphous form with low crystallinity that largely limits the transportation of photogenerated charge carriers. Therefore, it is necessary to search for other organic semiconductor materials with high crystallinity along with the presence of multiple active sites to further improve the photocatalytic efficiency.

### COF

**Figure 5:**
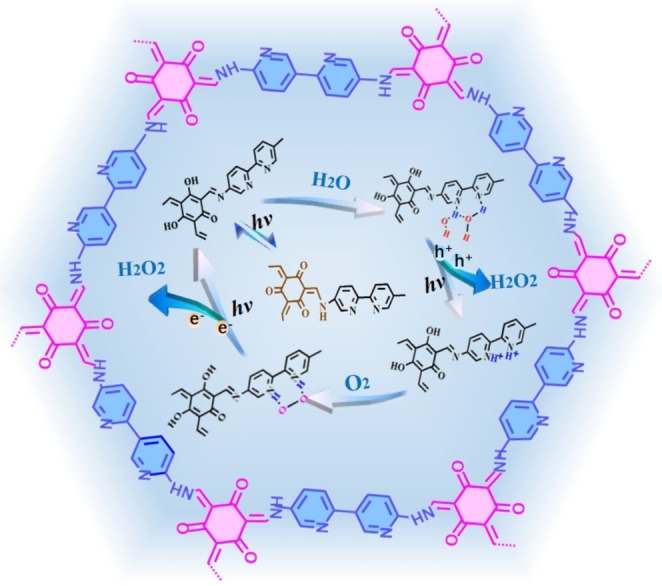
Crystalline COFs for H_2_O_2_ photosynthesis via 2e^−^ dual‐channels process.

Compared to other organic semiconductor materials, COF with high crystallinity has great advantages in the field of photocatalysis (Figure 5). It was firstly reported for H_2_O_2_ photosynthesis with ethanol as a sacrificial reagent in 2020.^[29]^ Recently, our research group developed bipyridine‐based COF photocatalysts that showed excellent activity for two‐channel photocatalytic synthesis of H_2_O_2_ with a superior SCC efficiency of 1.08 %.^[30]^ By comparing the monomer structure and the crystallinity of COFs, it was found that bipyridine serves as the main reactive site. Further theoretical study showed that COF‐TfpBpy (Tfp: 1,3,5‐ triformylphloroglucinol; Bpy: 2,2’‐bipyridine‐5,5’‐dia‐ mine) drove a one‐step two‐electron redox reaction to produce H_2_O_2_ whereas COF‐TfpDaaq (Tfp: 1,3,5‐ triformylphloroglucinol; Daaq: 2,6‐diaminoanthraquinone) altered the redox pathway to a two‐step single‐electron process by changing the active sites. In addition, based on in‐situ Fourier‐transform infrared (FTIR) spectroscopy, the high photoactivity of COF‐TfpBpy is attributed to the protonation of bipyridine monomer, which promotes the rate‐limiting reaction (2e^−^ WOR) and improves Yeager‐type oxygen adsorption to accelerate 2e^−^ one‐step oxygen reduction. In order to enhance the activity of COFs for H_2_O_2_ photosynthesis, highly polar ionic sites were introduced in COFs materials to promote the charge carrier separation.^[31]^ After ionization, the catalytic performance of COFs was increased by more than 5 times under visible light illumination.

Presently, most of the photocatalytic synthesis of H_2_O_2_ experiments were performed in a two‐phase system. However, the solubility of O_2_ in water is low (0.25 μmol cm^−3^ for oxygen‐saturated water), which greatly limits the H_2_O_2_ photocatalytic synthesis activity. In addressing this problem, three‐phase reaction interface was established with COFs, in which the H_2_O_2_ generated at the three‐phase interface was 2.9 mmol g^−1^ h^−1^,^[32]^ demonstrating a remarkable 15 times enhancement over the two‐phase system. That being said, the photocatalytic production of H_2_O_2_ from organic semiconductor materials is still in the nascent stage, and there is still much room for development. The search for suitable materials and efficient synthetic approaches to achieve stable and highly efficient photocatalytic production of H_2_O_2_ from pure water at high yields requires the joint efforts of researchers from different fields.

## Future Prospects

**Figure 6:**
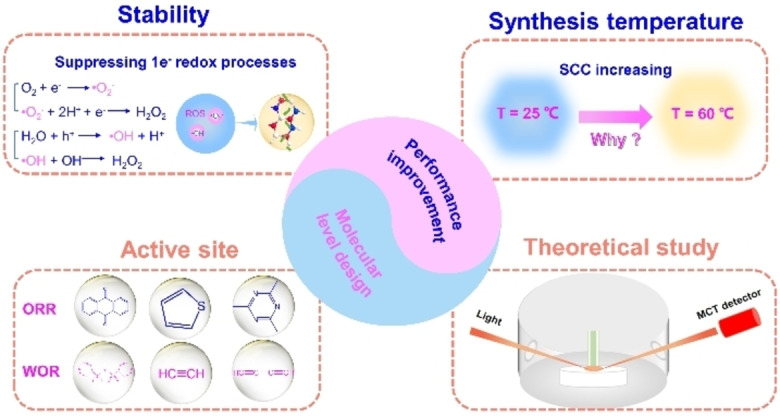
Key points for achieving remarkable pure H_2_O‐to‐H_2_O_2_ photosynthesis activity.

Researchers have basically reached a consensus in the field of photocatalytic synthesis of H_2_O_2_. In comparison to the inorganic materials for photocatalytic production of H_2_O_2_, organic polymeric semiconductor materials that do not contain any metal are more suitable for photocatalytic H_2_O_2_ production, and, ideally, the process should not involve any organic reagents as sacrificial reagents. This method has gradually developed into one of the most effective H_2_O_2_ synthesis methods with an aim of replacing the traditional anthraquinone oxidation. However, there is still a big chasm difficult to cross from laboratory extension to daily application. In order to solve this, a series of in‐depth studies from the aspects of molecular‐level design of organic polymeric semiconductor materials for improving the photocatalytic performance is necessary (Figure 6).

### Stability

Till date, most of the studies focus on the search for suitable photocatalysts or center on the modifications of the catalysts to improve the overall efficiency. However, the stability of the generated H_2_O_2_ and organic semiconductors are often neglected. It is worth mentioning that H_2_O_2_ can be decomposed by metal ions, UV light, heat, and the catalyst itself. For the H_2_O_2_ solution generated from the photocatalytic synthesis, there will be some residual organic catalysts inevitably present in the solution, which may lead to undesirable H_2_O_2_ decomposition. On the other hand, oxygen free radicals produced by the one‐electron pathway during photocatalysis will degrade organic polymers (⋅O^2−^ for oxygen reduction; ⋅OH for water oxidation). Therefore, to solve the problem ofstability, it is necessary to improve the stability of the material itself and the stability of H_2_O_2_ production. And improving the crystallinity of organic semiconductors, and then improve the efficiency of carrier separation of materials, the formation of multi‐electron double channel selectivity, to a greater extent to inhibit the generation of oxygen free radical, thus improving the stability of H_2_O_2_ production. Also increasing the degree of crystallinity of organic semiconductors will improve the stability of the material itself.

### Synthesis temperature

According to the empirical rule of Van't Hoff, the reaction rate increases by two‐to‐four times for every 10 K increase in the reaction temperature. The experimental studies demonstrated that the photocatalytic efficiencies increase gradually with the increase in temperature for both resins and COFs.^[25]^ However, the underlying mechanism is still unclear. For example, how the reaction temperature affects the adsorption of substrate molecules, the separation efficiency of charge carriers, and the activity of active sites still needs to be systematically investigated. If the temperature of the reaction system increases to 60 °C under the irradiation of sunlight, the photocatalytic performance is expected to increase by more than 50 %.^[30]^ Based on this phenomenon, the possibility of solar‐induced photothermal synergistic reaction for the synthesis of H_2_O_2_ could not be excluded. Therefore, visible to near‐infrared response activities of the organic semiconductors are equally deserving of much attention due to the potential photothermal effect.

### Active site

As mentioned previously, H_2_O_2_ can be obtained by oxygen reduction or water oxidation via photocatalytic reactions. There are three ways for the oxygen reduction or water oxidation: 1e^−^ process to reactive oxygen species (⋅O_2_^−^ for oxygen reduction; ⋅OH for water oxidation), 2e^−^ process to H_2_O_2_, and 4e^−^ process to either H_2_O (oxygen reduction) or O_2_ (water oxidation). Evidently, the dual‐channels 2e^−^ paths are more conducive to the generation of H_2_O_2_. However, there are still very limited organic semiconductor materials with dual‐channels 2e^−^ paths for the photosynthesis of H_2_O_2_. Since the difference in reaction pathways is closely related to the active sites in the catalysts, the active sites of different catalysts should be identified. For instance, the C=N (imine bond) in g‐C_3_N_4_, CTFs, and COFs is supposed to be the active site. Therefore, to improve the efficiency of photocatalytic generation of H_2_O_2_, it can be achieved by constructing more C=N bonds or investigating other possible active sites for O_2_ reduction, such as thiophene, anthraquinone, pentacenne‐dichromene‐dione, triazine, and benzene groups. Meanwhile, since the rate of WOR determines the overall performance of photocatalytic synthesis of H_2_O_2_, it is meaningful to introduce more active sites for WOR, such as acetylene, diacetylene, mellitic triimide, and perylene imides group.

### Theoretical study

The findings from the theoretical study can provide reliable technical supports for designing efficient and stable organic photocatalysts. In the process of photocatalytic synthesis of H_2_O_2_, in situ spectral characterization techniques and theoretical calculations should be the most effective methods. The in situ spectral characterization such as in situ IR and Raman spectroscopies were generally used to confirm the conformational changes of the catalysts and the intermediate species in the reaction. However, at present, gas–solid in situ technology is commonly used, which is far from the actual liquid–solid reaction environment. Therefore, with an aim of revealing the process of photocatalytic synthesis of H_2_O_2_ more accurately, liquid–solid in situ spectral technology should be introduced to understand the interaction mechanism between H_2_O and O_2_. In addition, theoretical calculations including band structures, differential charge density mappings, adsorption energies, and possible reaction pathways can also be used as supplementary evidence to support the results from in situ spectral characterization. With these, the reliability of the photocatalytic synthesis of H_2_O_2_ mechanisms will be greatly improved.

All in all, this perspective summarizes the current problems for photocatalytic synthesis of H_2_O_2_ over organic polymer semiconductors in terms of their stability, synthesis temperature, photocatalytic mechanisms, and active sites. In addition to the above‐mentioned aspects, there are still many factors waiting for the researchers to explore and study in order to develop an organic photocatalytic system with remarkable efficiency.

## Conflict of interest

The authors declare no conflict of interest.

1

## Data Availability

The data that support the findings of this study are available from the corresponding author upon reasonable request.
